# Neurological manifestations of COVID-19 – an approach to categories of pathology

**DOI:** 10.1186/s42466-021-00138-9

**Published:** 2021-07-26

**Authors:** Yana Leven, Julian Bösel

**Affiliations:** Department of Neurology, Klinikum Kassel, Mönchebergstraße 41-43, 34125 Kassel, Germany

**Keywords:** SARS-CoV-2, COVID-19, Neurological manifestations, Central nervous system, Peripheral nervous system, Standardised case definitions, Categories, Syndromes

## Abstract

**Background:**

Various neurological manifestations of infection with severe acute respiratory syndrome coronavirus 2 (SARS-CoV-2) have been reported, associated with a broad spectrum of diverse neurological symptoms and syndromes. Estimating rate and relevance of these manifestations remains difficult as there is a lack of standardised case definitions.

**Methods:**

We defined comprehensive categories including most reported neurological manifestations associated with SARS-CoV-2 to allow for a more standardised data collection. After a literature search of MEDLINE with ten keywords, 12 selected studies and larger case series were included. We compared the rate and relevance of neurological manifestations in hospitalized patients. We propose four main categories including 1) cerebrovascular disease, 2) inflammatory syndromes of the central nervous system (CNS), peripheral nervous system (PNS) and muscle, 3) metabolic/toxic dysfunction of CNS, PNS and muscle and 4) miscellaneous disorders.

**Conclusion:**

Ageusia (702) and anosmia (805) have been reported as the most common and the first occurring neurological symptoms. Cerebrovascular disease (451) and encephalopathy (663) were associated with a more severe course and worse clinical outcome. Any neurological manifestation was associated with a longer hospital stay and a higher morbidity and mortality compared to patients without neurological manifestations. We suggest reporting future neurological manifestations of coronavirus disease-19 (COVID-19) following a pathophysiology-based approach using standardized pre-defined case definitions to yield more specific and comparable data.

## Introduction

The crisis caused by SARS-CoV-2 has kept the world in suspense since its outbreak in Wuhan, China in 2019. What started as an epidemic was soon declared a pandemic by the World Health Organization (WHO) on the 11th of March 2020 [35, 36]. At the time of writing, 3.3 million COVID-19 deaths have been reported by the WHO and over 160 million cumulative cases globally since the start of the pandemic. Although respiratory disease caused by SARS-CoV-2 accounts for most of the severe courses and hospital admissions there has been emerging evidence on neurological manifestations of SARS-CoV-2. These neurological complications are of clinical relevance as they number among the first presenting symptoms and are associated with a more disease course.

In this narrative review we summarise the data on the distribution of neurological manifestations in hospitalized patients, associated with SARS-CoV-2 described in selected literature. We report the rate and relevance of neurological syndromes associated with the virus and suggest a pathophysiology-based approach for the future collection of data on neurological manifestations associated with SARS-CoV-2.

### Pathophysiology

SARS-COV-2 is part of the corona virus family comprising seven different virus types that are known to infect humans. It is a large enveloped positive strand-RNA virus with spike proteins on its surface appearing like a solar corona. These surface proteins are used to bind to the human angiotensin-converting-enzyme receptor 2 (ACE2) on human cells and the virus uses the transmembrane protease serine subtype 2 (TMPRSS2) to prime the spike proteins. Therefore, the presence of ACE2 on human cells determines viral cellular tropism. ACE2 is not only expressed in the lung parenchyma, kidney, pancreas, small intestine, testicles and vascular epithelia, but also in the CNS, including neurons and glial cells [37].

### Mechanism of CNS invasion

**Fig. 1:**
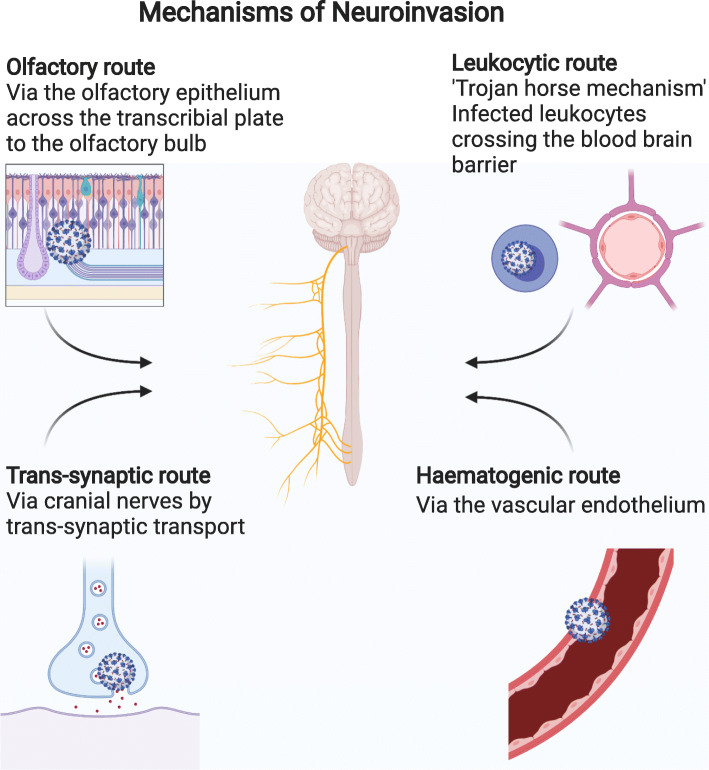
Mechanisms of Neuroinvasion (Created with BioRender.com)

Although SARS-CoV-2 primarily presents with respiratory features, several neurological manifestations have been described. This has initiated research on neurotropism of SARS-CoV-2 and resulted in evidence of its capability to infect nerve cells, adding it to a list of neuro-invasive coronaviruses. There are different routes via which SARS-CoV-2 is suspected to infiltrate the CNS including the olfactory route, the trans-synaptic route, the leukocytic route and the haematogenic route (summarised in Fig. 1). Other suspected routes of invasion also include the route via infection of the gastrointestinal tract as well as the vagus nerve [32]. Employing the first route described, the virus may spread directly via the olfactory nerve through the cribriform plate into the CNS, showing close clinical correlation given the anosmia seen as an early symptom of SARS-CoV-2 infection. The second route starts with an infection of peripheral nerves with resultant retrograde axonal transport of virus particles into the CNS. In the third route, virus particles travel to the CNS via the migration of infected leukocytes across the blood brain barrier (BBB). The fourth route is assumed to contain infection of and consequently the transport across vascular endothelium [26, 37].

### Mechanisms of CNS damage

**Fig. 2:**
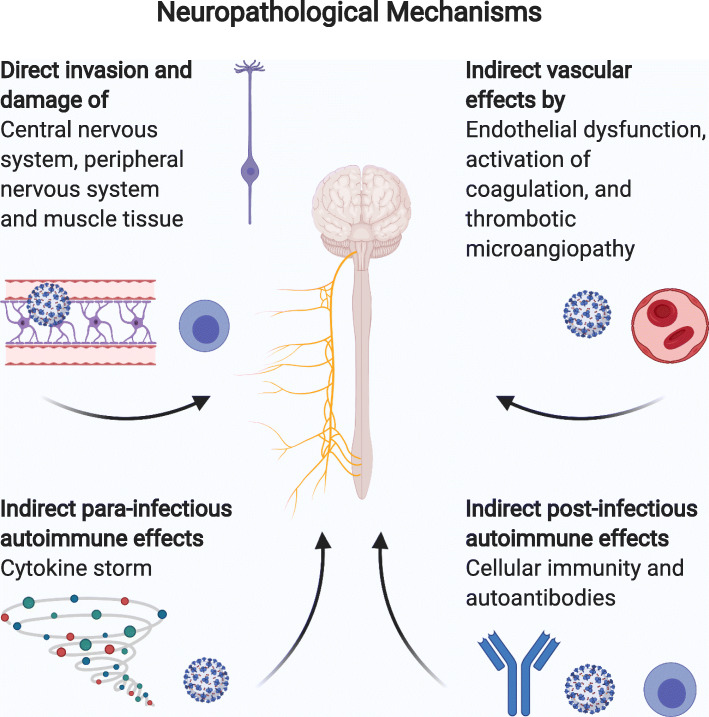
Neuropathological mechanisms (Created with BioRender.com)

SARS-CoV-2 is thought to affect the nervous system in different ways including direct and indirect neuropathological effects. These include direct damaging of the CNS, PNS and muscle tissue, indirect vascular effects, para-infectious autoimmune effects (cytokine storm) and post-infectious autoimmune effects (cellular immunity and autoantibodies) illustrated in Fig. 2 [3]. Panciani et al. postulate three phases of infection divided into neuroinvasion, CNS clearance and immune response. According to this pre-clinical model, the virus reaches the CNS via the haematogenous or olfactory route. Viral load is subsequently expected to rise within the cerebrospinal fluid (CSF) as viral replication takes place. During the phase of CNS clearance, viral load within the CSF is expected to decrease while the virus could be detected in the brainstem affecting the respiratory drive. In a post-mortem case series neuroinflammatory changes of the brainstem were one of the most common autopsy findings, with viral particles found within the medulla oblongata affecting the respiratory centre as well as cranial nerves emerging from the brainstem [17]. Finally, immuno-modulatory mechanisms trigger the production of antibodies against glial cells and neurons resulting in para- and post-infectious phenomena. In this phase the reciprocal interaction between the respiratory system and the CNS leads to neurotoxic hypoxia, worsening respiratory failure and subsequent hypoxic brain damage. Nevertheless this remains a pre-clinical model illustrating a possible path of pathogenesis to simplify the understanding of several possible mechanisms [20].

### Mechanisms of PNS and muscle affection

PNS and muscle can both be affected by direct invasion or systemic immune response. ACE2 is expressed in muscle tissue which presents one possible route of invasion. Peripheral nerves are directly affected and it is postulated that SARS-CoV-2 infection of nervous tissue induces downregulation of ACE2 leading to the overreaction of the Renin-Angiotensin-pathway resulting in neuroinflammation, oxidative stress and vasodilation [1]. Systemic immune response triggered by SARS-CoV-2 infection can result in PNS involvement by systemic hyperinflammation, macrophagic activation syndrome or also known as cytokine storm [18].

### Neurological manifestations of COVID-19

While numerous reports and reviews of neurological manifestations of SARS-CoV-2 have emerged to date, the comparability of data across study groups, institutions, and countries remains difficult as different reporting standards are used. Case definitions of neurological manifestations sometimes use a pathophysiology-based approach and at other times use a symptom-based approach, often mixing both approaches. Systematic, standardized case definitions as well as uniform diagnostic criteria of SARS-CoV-2 infections in general and nervous system infection/affection are lacking. Accordingly, overlaps, redundancies, and uncertainties as to which symptom is caused by what type of affection impedes the systemic evaluation of published reports. Without uniform and pre-defined case definitions, the prevalence, incidence, severity, and impact of neurological manifestations of SARS-CoV-2 remain challenging to ascertain. This complicates the development of diagnostic criteria, treatment guidelines and prognostication of neurological affection by SARS-CoV-2 infection.

To define neurological manifestations of COVID-19 requires the use of a standardized definition of the clinical association between neurological disease and SARS-CoV-2. The WHO has provided COVID-19 case definitions for confirmed, probable and suspected cases [35, 36]. These have been applied to neurological manifestations such as SARS-CoV-2 associated meningitis, encephalitis, myelitis, acute disseminated encephalomyelitis (ADEM), Guillain-Barré syndrome (GBS) and stroke by Ellul et al. [3]. Using these definitions across countries would increase the validity of data and could make the difference between a non-specific and specific association compensating for a lack of diagnostic evidence to exclude other causes.

In the following we propose definitions for conditions included in the main categories.

Cerebrovascular disease is defined as a neurologic symptom or syndrome caused by cerebral ischaemia or haemorrhage with a sudden onset of a focal neurological deficit [9].

Encephalopathy describes global brain dysfunction with different underlying pathologies such as metabolic, hypoxic-ischaemic, or septic pathogenesis. No uniform definition exists, and it is not accounted for in formal diagnostic manuals. This issue was addressed by a multi-society consensus paper generating a new recommendation on nomenclature. They define acute encephalopathy as a rapidly (in less than 4 weeks) developing pathobiological brain process expressed clinically as either subsyndromal delirium, delirium, or coma with other possible features such as seizures or extrapyramidal signs. By definition, other terms used to describe encephalopathy such as altered mental state, acute confusional state, acute brain dysfunction and acute brain failure should not be used interchangeably [27].

For delirium we suggest using the five diagnostic criteria as defined in the Diagnostic and Statistical Manual of Mental Disorders. These define delirium as a disturbance in attention that develops over a short period of time with a disturbance in cognition both not better explained by a pre-existing condition. There should also be evidence from the past medical history that the disturbance is a direct physiological consequence of a medical condition [2].

Coma is defined as a state of complete unwakefulness graded using diagnostic systems such as the Glasgow Coma Score [27].

Hypoxic ischaemic brain injury is used to describe diffuse brain injury because of transient anoxia or hypoxia [5].

For CNS and PNS inflammatory syndromes (Encephalitis, ADEM, Myelitis and GBS) we suggest using the definitions used by Ellul et a. They define SARS-CoV-2 meningitis, encephalitis, myelitis, or CNS vasculitis as SARS-CoV-2 detected in CSF or brain tissue or evidence of SARS-CoV-2-specific intrathecal antibody and no other explanatory pathogen or cause found. Furthermore, ADEM, GBS and other acute neuropathies associated with SARS-CoV-2 infection are defined as neurological disease onset within 6 weeks of acute infection; and either SARS-CoV-2 RNA detected in any sample or antibody evidence of acute SAR S-CoV-2 infection and no evidence of other commonly associated causes [3].

## Methods

We defined four categories that included the main neurological complications associated with COVID-19 so far. In the next step we conducted a literature search in order to extrapolate data for our new system of categorisation.

In an attempt to comprehensively appreciate reported observations while trying to reduce –not eliminate – redundancies, the following categories were defined; 1) cerebrovascular disease, 2) inflammatory syndromes of CNS, PNS and muscle, 3) metabolic/toxic dysfunction of CNS, PNS and muscle and 4) miscellaneous disorders. Both reported manifestations as well as symptoms and syndromes were assigned a category. If no assignment was possible the manifestation was grouped under miscellaneous disorders. Thereafter, a search was limited to English language manuscripts and to studies and case series with *n* > 30. The literature search was started in December 2020 using the following keywords: *neurological, neurologic, nervous system, brain, manifestations, complications, peripheral nervous system, central nervous system, COVID-19, and SARS-CoV-2.*

After scanning the abstracts for syndrome categories of neurologic manifestations we developed a table to group the different neurological manifestations using a pathology-based approach. We assessed the rate of neurologic manifestations in relation to the total rate of COVID-19 cases in the respective publication, if reported. Neurologic manifestations such as altered mental state were grouped under encephalitis or encephalopathy depending on the underlying cause. One prospective case series from Singapore described the additional category of autonomic nervous system dysfunction. As this has only been scarcely described in present literature it was not included as a separate category [14].

Cases were excluded if the manifestations were too unspecific such as ‘other peripheral disorders’, ‘headache’, ‘dizziness’ and ‘syncope’. We report the rate of neurological manifestations as percentage per total SARS-CoV-2 infections if recorded and the rate of neurologic manifestations per category as shown in Table 1. We describe the relevance in association with morbidity, mortality and prolonged hospital stay. As not all studies reported treatment effects or follow-up findings, data on outcome were expected to be incomplete and non- exhaustive.

**Table 1:** Overview of neurologic manifestations in COVID-19 from identified reports

**1. Miscellaneous disorders:**	1676
*Movement disorders: ataxia, hyperkinetic, hypokinetic*	62
*Dysautonomia (orthostatic hypotension)*	41
*Agitation*	40
*Confusion*	26
*Anosmia*	805
*Ageusia*	702
**2. Metabolic/toxic CNS dysfunction**	**1106**
Encephalopathy	663
*Altered level of consciousness*	138
Seizure	104
Hypoxic ischaemic brain injury	67
*Altered mental state*	47
*Diffuse corticospinal tract signs*	39
Psychosis	21
*Dysexecutive syndrome*	15
Delirium	11
Myelopathy	1
**3. Cerebrovascular disease**	451
Acute ischaemic stroke	351
Intracerebral haemorrhage	61
*Sudden neurological symptoms with likely vascular cause*	16
Transient ischaemic attack	12
Cerebral venous sinus thrombosis	7
Subarachnoid haemorrhage	3
Cerebral vasculitis	1
**4. Muscle inflammation/infection**	**378**
*Myalgia + signs of inflammation*	251
*CKaemia*	73
Rhabdomyolysis	54
**5. Metabolic/toxic muscle dysfunction**	**253**
*Myalgia*	160
*Myopathy*	47
*Fatigue*	46
**6. Metabolic/toxic PNS dysfunction**	**74**
Neuropathy	50
Critical Illness Neuropathy (ICU acquired weakness)	17
*Flaccid paralysis*	5
*Peripheral motor and/or sensory deficit*	2
**7. PNS inflammation/infection**	**70**
Guillain-Barré syndrome + variants	65
Miller-Fisher syndrome	2
Cranial and peripheral neuropathy	1
Brachial neuritis	1
Radiculitis	1
**8. CNS inflammation/infection**	**67**
Encephalitis	46
Acute disseminated encephalomyelitis	12
Myelitis	4
Meningitis 3	3
Virus encephalitis	2
TOTAL COUNTED CASES	**4075**

Diagnoses are written in roman and symptoms are written in *italics*

## Results

The literature search yielded 12 full text articles in English literature describing neurological manifestations and complications of SARS-CoV-2 including a rapid review (901 patients), retrospective as well as prospective observational case series (13,414 patients), cohort studies (137 patients) and surveillance studies (153 patients).

### Assessment of SARS-COV-2-infection and manifestation grades

**Fig. 3:**
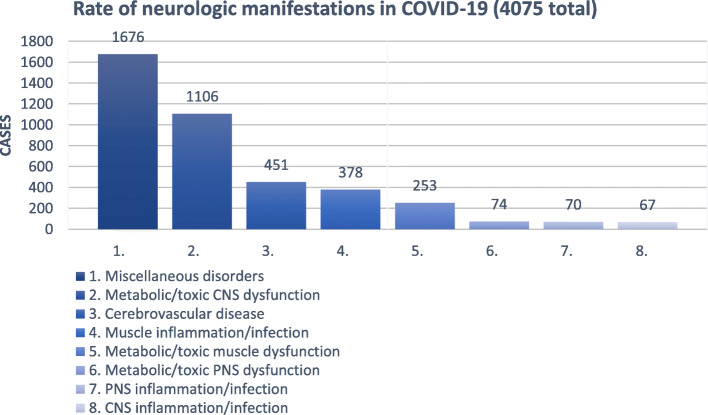
Rate of neurologic manifestations in COVID-19 (4075 in total)

All case series that were included identified a SARS-CoV-2 infection by a positive reverse transcriptase Polymerase Chain Reaction (rt-PCR) of SARS-CoV-2 from a nasopharyngeal swab. Additionally, some case series chose to utilize the WHO criteria for confirmed, suspected and probable cases. The infection severity of the SARS-CoV-2 associated pneumonia was graded using clinical scores such as the American Thoracic Society score for community-acquired-pneumonia. Stroke severity was sometimes assessed using the National Institutes of Health Stroke Scale and the degree of disability by using the Modified Rankin Scale. The Confusion Assessment Method for Intensive Care Unit was most often used to monitor delirium. Most studies reported clinical neurological, laboratory and radiological findings and, if examined, CSF analysis. Most also reported treatment approach of neurological manifestations associated with SARS-CoV-2 and, if recorded at the time, patient outcome at discharge (Fig. 3).

### Rate of neurological manifestations in SARS-CoV-2 patients

Our literature search identified 12 studies reporting categorizable neurologic manifestations in patients diagnosed with SARS-CoV-2. The overall rate of neurologic manifestations differed massively between studies. While four studies (Ellul et al., Paterson et al., Varatharaj et al. and Meppiel et al) did not compare the rate of neurologic manifestations in COVID-19 patients to those without neurologic manifestations, the remaining eight studies reported a rate ranging from 0.08% [14] to 90% [11]. Nearly all studies found more than one neurological symptom per patient, hence not all numbers add up to 100 %. Figure one displays the distribution of cases according to main categories of neurological manifestations. The most frequent neurologic manifestation reported was miscellaneous disorders with 1676 cases, mostly owing to the high frequency of anosmia and ageusia, followed by metabolic/toxic CNS dysfunction with 1106 cases and cerebrovascular disease being the third most common manifestation with 451 cases. The least common manifestation was CNS inflammation/infection with 67 cases. Table 1 shows the distribution between the main categories. Anosmia (805/1577 (51,05%)) and ageusia (702/1577 (44,51%)) were by far the most reported unspecific symptoms in the category of miscellaneous disorders. Encephalopathy (663/1106 (59,95%)) was reported most within the group of toxic/metabolic CNS dysfunction while neuropathy (50/74 (67,57%)) was most common within the group of toxic/metabolic PNS dysfunction. Within the category of cerebrovascular syndromes, acute ischaemic stroke (AIS) was the most common diagnosis (351/451 (77.83%)). Myalgia was the most frequent symptom within the category of muscle inflammation/infection (251/378 (66,40%) as well as toxic/metabolic muscle dysfunction (160/253 (63,24%)). Within the group of PNS inflammatory syndromes, GBS was the most frequent diagnosis (65/70 (92,29%)). For inflammatory CNS syndromes, encephalitis was the most frequent diagnosis (46/67 (68,66%)).

### Outcome of COVID-19 patients with neurological manifestations in general

COVID-19 patients with neurological manifestations had a higher disease severity and a worse prognosis. In a retrospective observational case study, any neurological symptom was associated with longer hospital stay (8 days versus 5 days). Encephalopathy and stroke were associated with more severe disease, based on the need for mechanical ventilation (7 cases overall, 5 had more severe disease, p: 0,022) [15]. Meppiel et al. report that COVID-19 severity was particularly severe or critical in the 102 patients (45.2%) with neurological manifestations [19].

This could be due to pre-existing morbidity as Frontera et al. describe that patients with neurologic disorders were older, male, white, hypertensive, diabetic, intubated and had higher sequential organ failure assessment scores (*p* < 0.05). Even after adjusting for confounders, COVID-19 patients with neurologic disorders had an increased risk of in hospital mortality (HR 1.38 95% CI 1,17-1,62) and a decreased likelihood of discharge home (HR 0,72, 95% CI 0,63-0,85, *p* < 0.001) [8]. These results are in keeping with Liotta et al. who report a 38% increased risk of in-hospital death and a 28% reduced likelihood of discharge home after adjusting for other factors [15]. In contrast, factors associated with a favourable outcome were younger age, female sex, absence of pre-existing neurological disorders and absence of severe COVID-19 disease [15]. However, within the group of neurological manifestations, anosmia and ageusia have been associated with a better prognosis, being inversely related to death in COVID-19 patients in an international cohort study [22].

### Associated laboratory parameters, diagnostics, and treatment approach

#### CVD

Certain case studies reported a significant association between CVD and abnormal coagulation parameters with raised D-dimers, fibrinogen, and prolonged Partial Thromboplastin Time in combination with thrombocytopenia [3, 14, 21, 23, 24]. Treatment approaches included single or dual antiplatelet therapy, prophylactic as well as therapeutic low molecular weight heparin (LMWH), new oral anticoagulants, thrombolysis, mechanical thrombectomy and external ventricular drain placement [3, 5, 14, 21].

#### Encephalitis

Inflammatory CNS syndromes were rarely reported. For encephalitis, the diagnosis was either based on pathology or clinical presentation. Several pre-existing diagnostic criteria were used such as encephalitis defined by clinical consensus criteria [10, 28], criteria of the Infectious Diseases Society of America and American Academy of Neurology guidelines [25, 29–31, 34]. Other studies used individual definitions such as ‘encephalopathy with evidence of inflammation in the CNS (CSF white cell count >5cells, protein >0.45 g/dl or magnetic resonance imaging (MRI) consistent with inflammation)’ [33], ‘altered mental status lasting >24h plus one of the following criteria: white blood count (WBC) in CSF >5/mm^3^, presence of compatible acute lesion on brain MRI, rt-PCR positive for SARS-CoV-2 in CSF’ [19]. Ellul et al. used the WHO case definition of SARS-CoV-2 associated meningitis, encephalitis and myelitis [35, 36]. To exclude other causative pathogens, some studies tested serum for Herpes-Simplex-Virus 1, Varizella Zoster IgM antibodies, Hepatitis A, B and C and Human-Immunodeficiency-Virus 1 and 2 and syphilis. CSF was tested for anti-neuronal antibodies (N-methyl-D-Aspartate receptor, myelin oligodendrocyte glycoprotein, aquaporin-4, leucine-rich glioma inactivated-1, glutamic acid decarboxylase) in some studies and bacterial cultures of the blood and CSF were also tested and found negative [3, 21]. In the case study by Meppiel et al. 2/21 cases of encephalitis had a positive rt-PCR for SARS-CoV-2 in the CSF, the rest is suggested to be of para-infectious cause rather than a results of direct viral neuropathogenicity as shown by microvascular lesions on brain MRI suggesting potential implication of COVID-19 associates endothelitis or coagulopathy [19]. Additionally, single case reports with CSF positive for SARS-CoV-2 are very rare given the large number of patients world-wide. Another cross-sectional study also supports the hypothesis that para-infectious pro-inflammatory effects of COVID-19 are primarily responsible for neurological affections [4].

Treatment approaches included corticosteroids, hydroxychloroquine, intravenous immune globulin (IVIG), convalescent plasma, interferon beta and several antiviral drugs (lopinavir, ritonavir, remdesivir, favipavir, acyclovir) [3, 14, 21, 23]. Single case reports also support the evidence on successful treatment with immunoadsorption or IVIG [13].

#### Inflammatory PNS syndromes

The most reported manifestation was GBS with 65 cases. The diagnosis of GBS was mainly based on existing diagnostic criteria such as the Hadden criteria or the Brighton diagnostic criteria. The diagnosis was verified by albumino-cytological dissociation as well as typical demyelinating patterns in the electromyography in several studies [3, 14, 19, 23]. Treatment approaches of GBS comprised IVIG, plasmapheresis and corticosteroids. In addition, antiretrovirals such as lopinavir and ritonavir were concomitantly used [3, 14, 19, 21, 23].

#### Muscle inflammation/infection

Several disorders were reported under the category of skeletal muscle inflammation including rhabdomyolysis (54/378), myalgia (251/378) and elevated Creatine Kinase (CK) levels in the serum (73/378). All studies reported a raised serum CK in association with raised C-reactive-protein (CRP), myoglobulin and lactate dehydrogenase.

#### Metabolic/toxic CNS dysfunction

Encephalopathy was defined differently in each study owing to the general lack of a standardized definition. Most definitions were symptom-based or partly symptom- and pathology-based, encompassing an alteration in the level of consciousness in the absence of signs of CNS inflammation [33]. Only two studies included the component of time, defining an encephalopathy as an altered mental status lasting over 24 h, associated with a seizure or focal neurological sign in the absence of encephalitis criteria, defined as COVID-19-associated if not accounted for by another toxic or metabolic cause [19] or as a pathobiological process that develops over hours to days and can manifest as changed personality, behaviour, cognition or consciousness [3]. Only Frontera et al. considered confounders such as sedatives, other drugs or hypotension as alternative causes of encephalopathic features in their definition of a COVID-19 associated encephalopathy [8]. Several symptoms were counted as presenting symptoms of encephalopathy such as seizures, altered mental state, altered level of consciousness or cognition, psychosis, delirium and pyramidal signs. As there are different aetiologies of encephalopathy it is difficult to validate the causality of a solely SARS-CoV-2 associated encephalopathy which could be seen in the context of a para-infectious inflammatory autoimmune reaction. If CSF or serum was examined for pathogens these were all found negative, including rt-PCR for SARS-CoV-2 [3, 21]. Laboratory parameters often showed abnormalities such as raised urea and nitrogen, acid-base disorders, electrolyte derangement and raised inflammatory markers (CRP, Procalcitonin) [3, 8, 15]. Neuroimaging was variably ranging from being normal (MRI/CT), over non-specific MRI changes to leptomeningeal enhancement. Coincidental findings of asymptomatic or previous strokes were also reported. If electroencephalography was performed it displayed diffuse bifrontal slowing or was unremarkable [3, 11, 21]. Treatment was largely supportive and symptomatic, and treatment approaches included corticosteroids, antiretrovirals, IVIG, LMWH and dialysis [3, 19, 21, 24].

### Relevance and outcome of selected neurological manifestations

#### CVD

Several studies point towards an association between CVD and more severe disease**.** Two studies reported a mortality of 15% [19] and 38.5% respectively [23] for SARS-CoV-2 positive patients with CVD. Fan et al. describe that AIS was associated with a longer period of hospitalization and higher mortality [5]. In a multicentre case study the rate of short-term mortality in COVID-19 patients with AIS was found as high as 15% (19/124). Nevertheless, they also report that five out of six patients improved or stabilized. In synopsis with the coagulopathy associated with COVID-19 infections this indicates that critically ill patients with COVID-19 and ischaemic stroke may benefit from anticoagulant therapy [5]. In contrast to that, in a prospective case series only two out of five patients with AIS had prothrombotic factors, and only 50% (2/4 patients) with cerebral venous thrombosis had demonstrable prothrombotic factors. Nevertheless, SARS-CoV-2 induced inflammation and endothelial dysfunction are likely contributory and they judge the excess mortality to be related to COVID-19 dysimmune coagulopathy [14].

#### Encephalitis

Outcomes were variable and often incomplete as most studies were published without follow-up data. One study reported a mortality of 4.8% and a recovery rate of 47% [19]. Ellul et al. found a mortality of 25% (2/8) but also a successful discharge home in 37.5% (3/8) of cases [3]. Out of four encephalitic patients, Koh et al. reported one who died (mortality 25%), three patients who did not respond to treatment and one who recovered [14].

#### Inflammatory PNS syndromes

In the rapid review of Ellul et al. outcomes were split with one third receiving physiotherapy, one third being discharged and one third still ventilated at the time of publication [3]. In a retrospective observational cohort study, immunoglobulin treatment shortened recovery and improved disability score when administered in the early stage of GBS [23]. Two of 15 patients (13.33%) required mechanical ventilation, one of these had the acute-motor-sensory-axonal-neuropathy variant of GBS [3]. No death in 15 patients was reported from a retrospective multicentre case series [19].

#### Muscle inflammation/infection

Treatment was reported as supportive in one study with good outcome as symptoms improved [3]. In contrast to that, Romero-Sanchez et al. found a longer stay in the intensive care unit (ICU) as an independent predictor in multivariate analysis (OR 1.3, 95% CI 1.02–1.71, *p* = 0.03). However, uncertainty remains as to more than one symptom per patient was counted and therefore the primary manifestation could have been a different one with rhabdomyolysis being the result on the initial presenting complaint or a complication of ICU stay [24].

#### Metabolic/toxic CNS dysfunction

Ellul et al. reported 49 patients with encephalopathy requiring an ICU stay and only one patient who did not. Liotta et al. described an overall longer hospital stay in patients with encephalopathy. In the prospective study of Frontera et al., encephalopathy was the most common diagnosis and especially common among patients with severe COVID-19 [8]. As to outcomes, Meppiel et al. reported a mortality rate of 14.8% and a recovery rate of 50.7%, while Paterson et al. reported a recovery rate of nearly 90% including partial recoveries.

Encephalopathy was associated with worse outcome due to multifactorial reasons. Fan et al. report that patients with delirium could not tolerate non-invasive mechanical ventilation and were therefore referred to the ICU for invasive mechanical ventilation [5]. Liotta et al. report worse functional outcomes, higher mortality and morbidity for encephalopathy independent of respiratory disease severity [15]. Helms et al. found that acute respiratory distress syndrome (ARDS) due to SARS-COV2 infection was associated with encephalopathy, prominent agitation, confusion, and corticospinal tract signs [11]. In a multicentre retrospective case series, the rate of short-term mortality in patients with COVID-19 associated encephalopathy was 14.9%. Meppiel et al. describe a high proportion of patients with encephalopathy having pre-existing neurodegenerative disorders which may reflect the fact that chronic cognitive impairment is a known risk factor for delirium in patients with acute illness [19].

## Discussion

In this review we categorized neurologic manifestations of COVID-19 as reported in larger case series and noteworthy studies. We found that neurologic manifestations are generally associated with a higher morbidity and mortality as well as a worse outcome. Even though the exact rate of neurologic manifestations in patients with COVID-19 remains unclear it is vital to raise clinical awareness in order to recognize and treat patients early and adequately.

One explanation for the high discrepancy between the rate of neurological manifestations in COVID-19 patients ranging from 0.08 to 90% could be the generosity of case descriptions. If cases were pre-defined the rate was naturally much lower than if cases were included based on a non-systematic subjective description derived from medical records. Secondly the rate of neurologic manifestations is strongly dependent on the population that is examined, such as in Helms et al’s cohort where only patients admitted to ICU due to severe ARDS where included in contrast to Koh et al’s cohort where all microbiologically confirmed COVID-19 patients in Singapore where included [11, 14]. We strongly recommend using case definitions such as created by Ellul et al. for SARS-CoV-2 meningitis, encephalitis, myelitis, or CNS vasculitis for future studies as these are the only standardised case definition for neurological manifestations associated with COVID-19 to date.

Even though PNS disorders including the presentation with anosmia and ageusia were quantitatively the most frequent manifestations, encephalopathies and cerebrovascular disorders are of more systemic relevance due to a higher association with morbidity and mortality. Encephalopathy in general is a misfortunately chosen term as by definition it means that there is a malfunction of the brain which is neither a pathophysiologic nor a clinical definition and its use certainly remains problematic. Whether anosmia and ageusia are always SARS-CoV-2-related remains questionable and until proven otherwise this generous association should be made with caution. The differentiation between local epithelial, PNS or CNS affection underlying these symptoms remains similarly challenging. Nevertheless, these were reported as the earliest neurologic symptoms of SARS-CoV-2 infections and any patient presenting with these features warrants investigation for the disease.

An important issue raised by a review on neurobiology of COVID-19 applies to the proportion of neuro-inflammatory syndromes that are caused by direct viral affection of COVID-19 versus those that are caused by para- or post-infectious effects e.g. coagulopathy or endothelial inflammation [6]. This is still an ongoing controversy as valid data cannot be extracted if one does not aim to differentiate between these phenomena. Especially for CVD, thrombosis caused by endotheliitis and coagulopathy induced by hyperinflammation are suggested as the responsible pathomechanisms. We see the need to systematically perform CSF examination with PCR for SARS-CoV-2 genome and search for antibodies against SARS-CoV-2 in patients with symptoms related to CNS involvement to assess the causality of SARS-CoV-2 associated CNS inflammatory syndromes. It is therefore desirable to design further neuropathological studies to improve our understanding of the underlying pathogenesis and to approach causality. One of the largest post-mortem case series showed that neuroinflammation in general was only mild with no evidence of CNS damage directly caused by SARS-CoV-2 suggesting that para-infectious effects play a more important role than direct viral affection of the CNS [17].

### Limitations

This review has several limitations. Considerable studies did not provide detailed enough data to compare distinct parameters across different studies which decreases the validity of such comparison. For example, the “break-through” publication on the topic by Mao et al. only provide little insight into their approach of categorization and describe several CNS manifestations such as cerebrovascular disease, dizziness, headache, impaired consciousness, ataxia and seizures as one group without differentiating between symptoms, syndromes and diagnoses [16].

Altered mental state was often accounted for as a presenting symptom and studies handled this very differently. Varatharaj et al. divided the presenting symptom of altered mental status between encephalitis, encephalopathy or neuropsychiatric disorders depending on the assumed underlying pathology [33]. Meppiel et al. grouped altered mental state under encephalopathy or encephalitis depending on the presence of signs of inflammation (elevated WBC in CSF or compatible lesions on MRI) [19]. Rifino et al. assigned altered mental state as a presenting symptom to subcategories such as encephalitis, necrotizing encephalitis and virus encephalitis depending on the assumed underlying pathology [23]. Our approach was rather based on the underlying pathology and not the presenting symptom. This may well have led to a loss of such cases during the rate assessment.

The retrospective multicentre case series by Meppiel et al. was one of the few studies that actively excluded other possible aetiologies besides SARS-CoV-2 for encephalopathy and clearly defined neurological manifestations a priori [19]. Likewise, the observational case study of Paterson et al. which states that cases for which a more likely alternative pathology was found were excluded from the analysis even though the exact diagnostic exclusion is not specified [21]. The other studies did not account for alternative pathophysiological mechanisms such as Mao et al., where it could not at all be distinguished whether neurologic manifestations were caused by direct viral affection, pulmonary disease or other organ damage [16]. This issue applied to all other studies where no other pathogen or antibody investigations were done to exclude alternative aetiologies. Studies that counted more than one symptom or syndrome per patient were especially difficult to include as symptoms and syndromes were counted double or triple [24]. This made it impossible to extrapolate accurate numbers and only count valid diagnoses. Frontera et al. counted both new and existing neurological conditions and hence these could not be differentiated [8]. Finally, for the sake of specificity we completely ignored non-specific symptoms such as headaches, dizziness, nausea, etc. although some or even many of these may have reflected neurologic manifestations.

### Open questions and considerations for future studies

This narrative review offers an approach to categorization of neurological manifestation based on several big case series. Robust estimation of the situation is strongly affected by discrepancies in case definitions, mixing symptoms/syndromes with diagnostic findings, differences in patient cohorts, and lack of systematic exclusion of alternative causes of neurologic disease. Further prospective studies are needed to better understand the underlying pathophysiology of neurological manifestations in COVID-19 disease including case control studies to ascertain the actual rate and relevance of such manifestations. These studies need systematic definitions of manifestations such as those proposed by us or used in very few existing studies [8, 19]. Furthermore, it would be desirable to collect additional data on outcome parameters such as ICU need, length of stay, ventilation need and duration, mortality, cause of death, outcome at discharge as well as discharge destination as already implemented by the NCS Global Consortium Study or by young neurologists who collect data from the Lean European Open Survey on Sars-CoV-2 Infected Patients on neurological manifestations [7, 12].

## Conclusion

In conclusion, all reviewed studies demonstrate that neurological manifestations are broad and heterogenous suggesting different underlying pathogenic processes and pathways. It is crucial to explore the causality of SARS-CoV-2 regarding neurological manifestations and to exclude likely alternative causes. Therefore, clearly defined, standardized, and universal case definitions should be used across countries. Even though neurologic manifestations might represent a relatively small part of COVID-19 phenomena, early recognition and treatment is key to prevent a more severe outcome associated with higher morbidity and mortality.

## Data Availability

Not applicable.

## Abbreviations

ACE2: Angiotensin-converting-enzyme receptor 2
ADEM: Acute disseminated encephalomyelitis
AIS: Acute ischaemic stroke
ARDS: Acute respiratory distress syndrome
BBB: Blood brain barrier
CK: Creatine kinase
CNS: Central nervous system
COVID-19: Coronavirus disease 2019
CRP: C-reactive protein
CSF: Cerebrospinal fluid
CT: Computer tomography
GBS: Guillain-Barre Syndrome
IVIG: Intra-venous immune globulin
LMWH: Low molecular weight heparin
MRI: Magnetic resonance imaging
PNS: Peripheral nervous system
Rt-PCR: Reverse transcriptase polymerase chain reaction
SARS-CoV-2: Severe acute respiratory syndrome coronavirus 2
TMPRSS2: Transmembrane protease serine subtype 2
WBC: White blood count
WHO: World Health Organization
